# In Vitro and In Vivo Control of Secondary Bacterial Infection Caused by *Leishmania major*

**DOI:** 10.3390/ijerph14070777

**Published:** 2017-07-13

**Authors:** Hany M. Yehia, Ebtesam M. Al-Olayan, Manal F. El-Khadragy, Dina M. Metwally

**Affiliations:** 1 Department of Food Science and Nutrition, College of Food and Agriculture Sciences, King Saud University, Riyadh 11451, Saudi Arabia; 2 Department of Food Science and Nutrition, Faculty of Home Economics, Helwan University, Cairo 11221, Egypt; 3 Zoology Department, Faculty of Science, King Saud University, Riyadh 11495, Saudi Arabia; eolayan@ksu.edu.sa (E.M.A.-O.); manalelkhadragy@yahoo.com (M.F.E.-K.); mdbody7@yahoo.com (D.M.M.); 4 Chair Vaccines Research of Infectious Diseases, King Saud University, Riyadh 11495, Saudi Arabia; 5 Zoology Department, Faculty of Science, Helwan University, Cairo 11790, Egypt; 6 Parasitology Department, Faculty of Veterinary Medicine, Zagazig University, Zagazig 12878, Egypt

**Keywords:** bacteria, cutaneous leishmaniasis, *Leishmania major*, histopathological analysis, ciprofloxacin

## Abstract

Bacterial infections of cutaneous leishmaniasis cause skin ulcers on mice, resulting in increased tissue deterioration, and these infections can be controlled with liquid allicin. To isolate and identify the incidences of real secondary bacterial infections in mice, we performed the current study by injecting mice (*n* = 50) with *Leishmania major. L. major* infections were initiated by an intramuscular injection of 0.1 mL Roswell Park Memorial Institute (RPMI 1640 media/mouse (10^7^ promastigote/mL)). Scarring appeared 2–6 weeks after injection, and the bacteria were isolated from the skin ulcer tissues. Allicin (50 µL/mL) and ciprofloxacin (5 μg; Cip 5) were used for controlling *L. major* and bacteria. One hundred samples from skin ulcers of mice were examined, and 200 bacterial colonies were isolated. Forty-eight different genera and species were obtained and identified by Gram staining and physiological and biochemical characterization using identification kits. All samples were positive for secondary bacterial infections. Of the isolates, 79.16% were identified as Gram-negative bacteria, and 28.84% were identified as Gram-positive bacteria; only one yeast species was found. Interestingly, pure allicin liquid at a concentration 50 µL/mL exhibited antibacterial activity against a wide range of Gram-negative and some Gram-positive bacteria, in addition to yeast, and was 71.43% effective. Antimicrobial resistance patterns of all genera and species were determined using 15 different antibiotics. Allicin (50 µL/mL) and Cip 5 were the most effective against *L. major* and 92.30% of isolated bacteria. *Stenotrophomonas maltophilia* was the most resistant bacterium to the tested antibiotics with a survival rate of 73.33%, and it exhibited resistance to allicin.

## 1. Introduction

Cutaneous and visceral leishmaniasis are the most common forms of leishmaniasis present in Asia. Leishmaniasis occurs in 88 different countries, with 367 million persons at risk of infection [1] and at least 12 million contracting the disease. The incidence of cutaneous leishmaniasis is approximately one million per year, and the incidence of visceral leishmaniasis is 500,000 cases per year, according to recent estimates [2,3]. Approximately 90% of cutaneous leishmaniasis patients are in Afghanistan, Iran, Saudi Arabia, Brazil, and Peru [4]. The vector is primarily transmitted and spread through sand fly bites. The disease mechanisms responsible for healing or chronicity of experimental and human leishmaniasis are essentially confined to the immune system [5,6]. Bacterial, fungal, viral, and parasitic infections sometimes result and affect the integrity of the skin and immune system [7,8,9].

When scars caused by the infection of *Leishmania* parasites ulcerate, they become susceptible to microbial colonization from pathogenic bacteria and yeast, possibly stimulating secondary infections. Some authors have identified *Aerococcus viridans* in the environment and occasionally isolated it from human skin [10,11]. A previous report on the infection of immunodeficient mice identified *A. viridans* [12], which constitutes 5–10% of the bacterial flora in the air and dust in occupied rooms [13]. In addition, *A. viridans* has been found in small numbers in the upper respiratory tract and on the skin of healthy persons. *Chryseobacterium meningosepticum* has been found as an infection of skin, soft tissue, and wounds [14].

*Comamonas acidovorans* (*Pseudomonas acidovorans)* has been isolated from inhalation equipment and clinical sources, including cerebrospinal fluid, sputum, urine, the pharynx, and wounds, but was considered nonpathogenic in these cases [15]. Although *Chryseomonas luteola* has rarely been reported as a human pathogen, it has been known to cause meningitis septicemia, peritonitis, or endocarditis in patients with health or indwelling disorders [16,17]. *Burkholderia cepacia* and *Burkholderia gladioli* are plant, animal, and human pathogens and are common agents in nosocomial infections [18,19]. Members of this genus are also found in various ecological niches.

*Cryptococcus albidus* is a non-neoformans species and a rare cause of disease [20]. Cases of systemic disease in which this opportunistic yeast has been isolated from the lung, cerebrospinal fluid, and blood have been reported [20]. These studies also reported the first case of cutaneous *Cryptococcus* infection due to the species *Cryptococcus albidus*, found in a man with Sézary syndrome [20].

Allicin is a component of garlic (*Allium sativum*) that mediates its antibacterial effects [21]. Various bacteria and clinical isolates are sensitive to pure preparations of allicin [22]. In contrast, some bacterial strains, such as the mucoid strains of *Pseudomonas aeruginosa, Streptococcus hemolyticus*, and *Enterococcus faecium*, are resistant to the action of allicin. The reasons for this resistance are unclear. The hydrophilic capsule or mucoid layers are assumed to prevent the penetration of allicin into the bacteria; however, this hypothesis has yet to be studied in depth [23].

Anthony et al. [24], mentioned that garlic is one of the oldest plants used as a medicine; it has been considered a valuable healing agent by many different cultures for thousands of years. Sulfur compounds of the plant, such as allicin, diallyl trisulphide, and ajoene, can reduce the development of different protozoan parasites. The human intestinal protozoan parasite *Entamoeba histolytica* is very sensitive to allicin, and 30 μg/mL allicin completely inhibits growth of the amoeba cultures [25]. Similarly, 30 μg/mL allicin inhibited the growth of other protozoan parasites, such as *Giardia lamblia, L. major, Leptomonas collosoma,* and *Crithidia fasciculata* [26]. Some allicin toxicity has been observed in tissue-cultured mammalian cells at concentrations above 100 μM. However, no damage to the mammalian cells was noticed at higher concentrations of allicin in the presence of amoebic trophozoites; this discrepancy may be due to the affinity of the allicin molecules to the parasite targets [26]. Metwally et al. [27] demonstrated, for the first time, that allicin displays an antileishmanial effect under in vitro and in vivo conditions.

The aims of the present study were to investigate and identify common bacteria that cause skin ulcers in mice treated with *L. major* and determine the effects of allicin and different types of antibiotics on these bacteria.

## 2. Materials and Methods

### 2.1. Freezing Leishmania Major Promastigote

**Figure 1:**
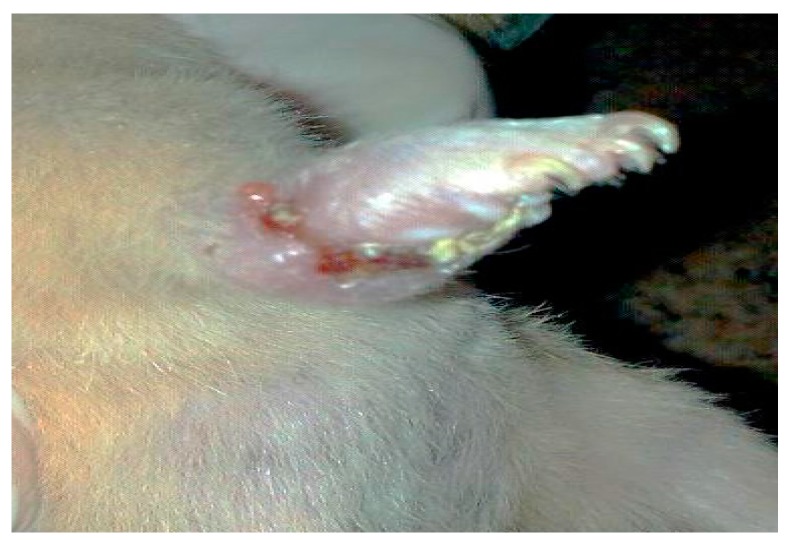
Lesion caused by *Leishmania major* infection in a female hamster right foot pad.

The suspended *L. major* promastigotes (Zymowme LON4, Riyadh, Saudi Arabia) of a Saudi sub-strain were aspirated from three-day-old culture flasks into 15 mL tubes. The cells were first counted using the four marginal squares of a hemocytometer (Sigma Aldrich Z359629, St. Louis, MO, USA), then spun down at 2500 rpm for 10 min. The sediment parasites were suspended in freezing medium (6 mL Fetal Bovine Serum (FBS), 3 mL RPMI 1640 and 1 mL dimethyle sulfoxide (DMSO)) then transfered into cryogenic vials. The vials were frozen in a −80 °C freezer for 48 h. The vials were subsequently transferred into liquid nitrogen containers. Isolation of Leishmania major from the right foot pad of the hamster was inoculated with 10^7^ of stationary phase (eight days old) *L. major* suspended in 0.1 mL RPMI medium. The hamsters were kept at the animal house for 3–4 weeks. Bagg Albino genotype c (BALB/c) mice were inoculated with *L. major* in the foot pad, forming a lesion that was then homogenized after sacrificing the animal (Figure 1). The animal was suffocated by using CO_2_ in a special container. The infected foot pad was then cleaned by using 70% ethanol before chopping it off. The foot pad was cleaned of skin, hair, and nails by using a sterile scalpel, forceps, and scissors. The flesh was moved into the homogenizing apparatus, placed in ice, and then 10 mL of complete RPMI 1640 medium was added. The medium was mixed with the flesh, which was smashed by continuous movement of the apparatus. The supernatant was transferred into a 15 mL tube and then spun again at 500 rpm for 5 min to sediment the remaining flesh and cells. The pellet was discarded and the supernatant was poured into a new 15 mL tube and spun at 2500 rpm for 15 min to sediment the parasite. In order to wash the isolated parasites, 10 mL of RPMI 1640 medium was added to the parasite pellet. Cells were re-suspended by a gentle vortex and the tubes were spun at 2500 rpm for 15 min. The supernatant was discarded and then 5 mL of RPMI 1640 medium was added to the tube. After vortexing the tubes gently, the mix was poured into two tissue culture flasks containing a previously prepared RPMI 1640 medium, and incubated at 26 °C for three days. Then, *L. major* promastigotes (ZymowmeLON4) were frozen in liquid nitrogen.

### 2.2. Preparation of the Dose of the Parasite to Inject Animals

After cultivation of *L. major* in RPMI 1640 and arriving at the stationary phase ready to be used to infect healthy animals, the medium was transferred into a 15 mL tube and then spun at 2500 rpm for 10 min. The supernatant was discarded and 10 mL of phosphate buffered saline (PBS) was added to the pellet. A hemocytometer slide was used to count living promastigotes using a normal lens magnification microscope (Carl Zeiss GmbH, Jena, Germany), at 40×, according to mathematical equation: the number of parasites/number of squares ×10^7^.

### 2.3. Experimental Animals

Female BALB/c mice weighing 15–21 g were obtained from the Animal House of King Saud University, Faculty of Sciences (Riyadh, Saudi Arabia). The animals were kept in wire bottomed cages in a room under standard conditions of illumination with a 12 h light-dark cycle 55 + 5% relative humidity and at 25 °C ± 2 °C for one week until the beginning of treatment. They were provided with tap water and a balanced diet ad libitum. All animals have received human care in compliance with the state authorities following the Saudi Arabia rules of animal protection.

### 2.4. Allicin Preparation and Concentration

Allicin was obtained as liquid Allisure at a concentration of 1000 mg/L from Allicin International Ltd. (Rye, East Sussex, UK). Disc filter paper (5-mm diameter) was saturated with 50 µL allicin for use as in antimicrobial activity.

### 2.5. Infection in BALB/c Mice

Animals were shaven in the deficit area using a sterile shaving machine by ethyl alcohol 70% and *L. major* infections were initiated by subcutaneous injection of 0.1 mL of RPMI 1640 media (each mL of media has 10^7^ promastigotes/mL) per mouse. Parasitemia was determined every other day by observing the lesion’s appearance (2–6 weeks post infection). After four weeks from the date of the injection (the incubation period), lesions started to appear with simple swelling (scar) and then gradually grew ulcerous, and the development of ulcers was followed up by using the length measurement (micrometer).

### 2.6. Isolation and Identification of Bacteria and Yeast

Using cotton wool moistened with alcoholic iodine, the area of the skin surrounding the lesions was cleaned thoroughly. After appropriate cleaning, the ulcer specimens were obtained by rubbing sterile saline solution over the edge of the ulcerated lesions and then transferring the swabs to Muller Hinton broth and nutrient broth medium. After a 24 h incubation at 37 °C, one loop of the samples was streaked on each of Muller Hinton Agar, Nutrient agar, M-Enterococcus agar, Ogwa agar, and Sabouraud agar. Gram staining was used to determine the species of bacteria.

API 20 E, API 20 NE, API 20 STREP, and API 20C (BioMérieux, Marcy l’Etoile, France) were used for the identification of different isolates. The strips were inoculated, incubated at 37 °C for 24 h or 48 h, and analyzed according to the manufacturer’s instructions for each kit. The reactions were recorded, and the identifications were determined using a computer program (API Lab Plus software version 3.2.2 (BioMérieux, Marcy l’Etoile, France)).

### 2.7. Antimicrobial Susceptibility

As previously described [28], the antimicrobial susceptibility test for each isolate was performed for freshly-prepared samples of different genera and species. The overnight cultures of tested bacteria (inoculated from a single colony) were freshly grown for 4 h at approximately 10^6^ Colony Forming Unit (CFU/mL) on dry surfaced Mueller Hinton agar (Oxoid Limited, Basingstoke, UK) using the agar disk diffusion method [28]. A total of 15 antibiotic discs (Oxoid) were tested, including 25 μg ampicillin (AML 25), 30 μg neomycin (N 30), 75 μg ticarcillin (TIC 75), 30 μg vancomycin (VA 30), nitrofurantoin (F 300), 30 μg linezolid (LZD 30), 25 μg sulfamethoxazole trimethoprim (SXT 25), 25 μg colistin sulfate (CT 25), 5 μg ciprofloxacin (Cip 5), 30 μg kanamycin (K 30), 30 μg chloramphenicol (C 30), 30 μg tetracycline (TE 30), and 30 μg cefadroxil (CFR 30). The diameters of the zones of inhibition (mm) were interpreted using the criteria recommended by the NCCLS [29], and the isolates were classified as either sensitive (S) or resistant (R) strains. The results were interpreted using the sizes of the inhibition zones based on the measured diameters [30,31,32].

### 2.8. Effect of Allicin Liquid on Bacterial Isolates

Disc filter paper (5-mm diameter) was saturated with 50 µL allicin and placed on the surface of Muller Hinton Agar medium (Oxoid, CM0337) inoculated by the tested microorganisms 100 µL of 10^6^/mL. After incubating 24 h at 37 °C, the zone of inhibition (mm in diameter) was used to measure the inhibitory effect of allicin liquid on bacterial isolates. In addition, the mice were dosed with 50 µL allicin liquid via oral gavage.

### 2.9. Experimental Protocol (In Vivo)

Fifty female BALB/c mice were allocated randomly to five experimental groups (*n* = 10 mice/group) as follows:

- Group l: Normal non-infected negative control group.
- Group 2: Infected non-treated positive control group: Mice were inoculated subcutaneously with a dose of 0.1 × 10^7^ promastigotes in a shaved area above the tail.
- Group 3: Infected mice treated with liquid allicin at concentrations of 0.30 μM/mouse starting with the first appearance of ulcerative lesion. Treatment was continued for four weeks.
- Group 4: Infected mice treated with ciprofloxacin (Cip, 10 mg/mL) with the first appearance of ulcerative lesion. Treatment was continued for four weeks.
- Group 5: Infected mice were treated with 0.30 μM of liquid allicin and concomitantly with the antibiotic ciprofloxacin (Cip, 10 mg/mL) with the first appearance of ulcerative lesions. Treatment was continued for four weeks.

Treatment was initiated when local lesions were apparent. The mice were treated daily for four continuous weeks. Each week, the lesion size was measured before and after treatment with Vernier calipers. Parasitaemia was determined every other day by observing the lesion’s appearance (3–4 weeks post infection). Mortality was checked daily. All experiments were performed in compliance with the local animal ethics committee improvement requirements. Effects on ulcerative lesions were assessed clinically. The cure for the ulcers was defined as clinical according to the reduction in the size of the lesions compared with untreated infected control mice. The lesion size was calculated using the following formula:

Lesion Size = a + b/2

### 2.10. Histopathology

Using Giemsa and hematoxylin–eosin (H-E) staining fragments after being fixed in 10% buffered formalin, then embedding in paraffin.

### 2.11. Ethic Statement

All experiments were performed in compliance with local animal ethics committee requirements. We followed the European Community Directive (86/609/EEC) and national rules on animal care in accordance with the NIH Guidelines for the Care and Use of Laboratory Animals 8th Edition. The study protocol was approved (no. 2/3/12337) by the Ethical Committee of King Saud University (KSU), Riyadh, of the joined work between the College of Science (KSU) and the Zoology Department (Helwan University).

## 3. Results

**Figure 2:**
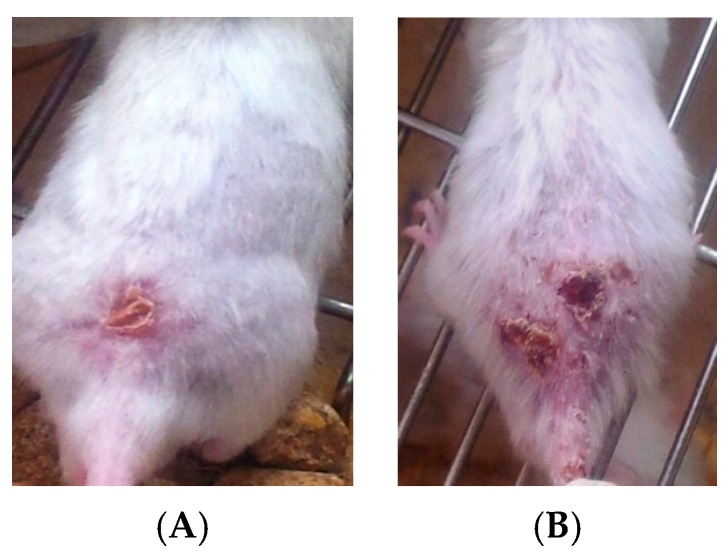
(**A**,**B**) Cutaneous leishmaniasis scars of Bagg Albino genotype c (BALB/c) mice appeared on the skin of mice four weeks after injection.

Clinically, cutaneous lesions in all infected groups started with redness and swelling at the site of inoculation on the third week of the infection. Swelling increased progressively, and crust formation occurred, with gangrene starting to develop by the fourth week of infection, as shown in Figure 2A,B. The lesions were measured twice a week by using a special scale bar (micrometer), and their means were calculated. The mean thickness of infected control mice increased gradually to a width of 15.7 mm ± 1.63 mm and a length of 16.67 mm ± 0.99 mm at the seventh week of the experiment.

**Figure 3:**
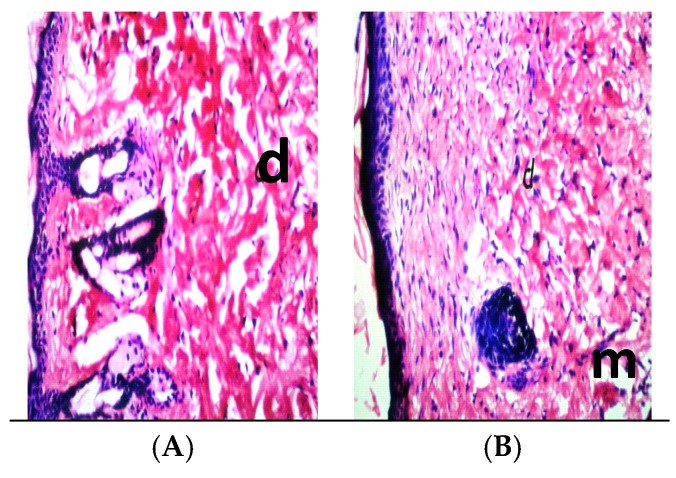
(**A**,**B**) Light micrograph (LM) of skin-infected mice at four weeks post-infection with 0.1 mL of 10^7^/mL promastigotes of *Leishmania major*, d: showing the intact histological structure of the epidermis and dermis, and a very large amount of inflammatory cell infiltration and fibroblastic proliferation in the subcutaneous layer (m).

Examination of H-E staining of naïve BALB/c mice of the infected non-treated mice sacrificed at four weeks post-infection identified a very large amount of subcutaneous infiltration of inflammatory cells and proliferation in the subcutaneous layer (m) (Figure 3A,B).

Results in Table 1 show the effect of liquid allicin (0.30 μM/mouse) on the lesions caused by *L. major* reduced to 5.65 mm, while through the use of Ciprofloxacin (10 mg/mL) reached 6.79 mm and, when used together, reached 3.17 in comparison to the control group, which measured 10.8 mm.

Data in Table 2 shows bacteria and yeast which were isolated from all of the skin ulcers on different specific media. Most isolated bacteria (38 isolates, 79.16%) were Gram-negative, whereas Gram-positive bacteria (10 isolates) comprised 20.84% of the isolates. *Flavimonas oryzihabitans* was the mean highest Gram-negative bacteria with six isolates (12.5%), followed by four isolates that were identified as *Chryseomonas luteola*, *Burkholderia cepacia*, *Aeromonas salmonicida* subsp. *salmonicida*, and *Chryseobacterium meningosepticum* (8.33%). Two isolates (4.16%) were identified as containing *Erwinia* sp., *Pantoea* sp., *Stenotrophomonas maltophilia*, *Pseudomonas aeruginosa*, *Aeromonas hydrophila*, *Flavobacterium indologenes*, *Chryseomonas indologenes*, and *Comamonas acidovorans*. Only two isolates were identified as *Cryptococcus albidus*. *Mycobacterium ulcerans* did not appear on Ogwa agar medium.

Fewer Gram-positive isolates were found than Gram-negative isolates, including four isolates of *Aerococcus viridians* (4.16%) and two isolates each of *Bacillus* sp. and *Lactococcus lactis* subsp. *lactis*, totaling 8.33%. Only two isolates of *Cryptococcus albidus* were found (4.16%).

Table 3 shows that all bacterial isolates were affected by 50 µL/mL allicin liquid except *Flavimonas oryzihabitans*, *Erwinia* sp., *Stenotrophomonas maltophilia*, and *Comamonas acidovorans.* The greatest effect of allicin liquid was observed for *Aeromonas salmonicida* subsp. *salmonicida*, *Pantoea* sp., and *Bacillus* sp., in which an inhibition zone of 15 mm was found; the next greatest effects were observed as a 12-mm inhibition zone for *Chryseobacterium meningosepticum* and 10-mm inhibition zones for *Chryseomonas indologenes*, *Burkholderia cepacia*, and *Pseudomonas aeruginosa* [24].

Table 4 shows that most (92.30%) bacteria were highly sensitive to Cip, except for *Flavimonas oryzihabitans*, which appeared to be resistant to it, accounting for 7.70% of the isolates. CT 25, C 30, and TE 30 comprised 84.61% of the isolates, but two isolates (15.39%) were resistant for each antibiotic, as follows: *Chryseomonas indologenes* and *Flavimonas oryzihabitans* for CT 25, *Erwinia* sp. and *Chryseomonas luteola* for C 30, and *Burkholderia cepacia* and *Pseudomonas aeruginosa* for TE 30. SXT 25 affected all but three bacterial strains (76.92% sensitivity rate), which were *Erwinia* sp., *Pantoea* sp., and *Stenotrophomonas maltophilia* (comprising 23.08%). N 30 and E 15 affected all but six bacterial strains, which were *Comamonas acidovorans*, *Chryseomonas indologenes*, *Flavimonas oryzihabitans*, *Burkholderia cepacia*, *Pseudomonas aeruginosa*, and *Stenotrophomonas maltophilia* for N 30 and *Comamonas acidovorans*, *Erwinia* sp., *Burkholderia cepacia*, *Pseudomonas aeruginosa*, *Aeromonas hydrophila*, and *Stenotrophomonas maltophilia* for E 15, with an overall resistance rate of 46.15%. AML 25, AMP 25, VA 30, and F 300 each affected five bacterial isolates (38.46% sensitivity rate), and eight isolates were resistant for each antibiotic (61.54%).

TIC 75 and K 30 affected all bacterial strains (61.54%) except for five isolates that were resistant (38.46%). These isolates were *Chryseomonas luteola*, *Chryseomonas indologenes*, *Flavimonas oryzihabitans*, *Pseudomonas aeruginosa*, and *Stenotrophomonas maltophilia* for K 30 and *Comamonas acidovorans*, *Chryseomonas indologenes*, *Burkholderia cepacia*, *Pseudomonas aeruginosa*, and *Stenotrophomonas maltophilia* for TIC 75. Most of the bacterial isolates were resistant to LZD 30 (76.92%), whereas the rate of sensitivity was 23.06%, including *Chryseobacterium meningosepticum*, *Bacillus* sp., and *Chryseomonas luteola*. CFR 30 affected four bacterial isolates (30.76%), and all other isolates were resistant (53.8%).

**Figure 4:**
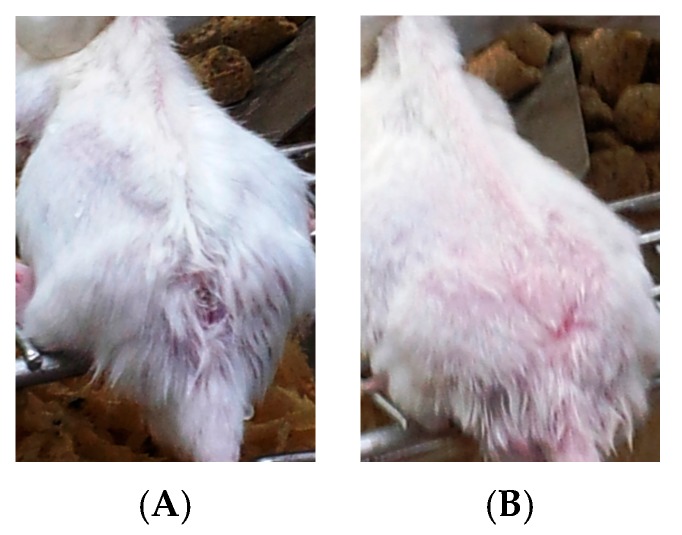
(**A**,**B**) Cutaneous leishmaniasis scars of BALB/c mice disappeared after treatments (four weeks post infection), with allicin and Cip.

**Figure 5:**
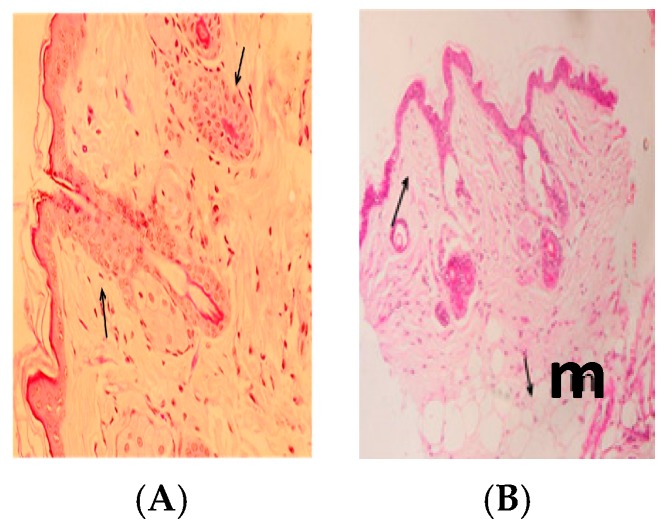
(**A**,**B**) LM of skin infected treated mouse at four weeks post-infection with 0.1 mL of 10^7^/mL promastigotes of *Leishmania major*, showing normal histological structure of epidermis and dermis. Arrows point to inflammatory cell infiltration in dermal and subcutaneous tissue (m) H-EX40.

Figure 4A,B show that the ulcerated skin appeared nearly normal after treatments with allicin and Cip. In addition, the histopathological analysis of the skin appeared normal in the epidermis (p) and dermis (d) tissues (Figure 5A), whereas Figure 5B shows little inflammatory cell infiltration in the subcutaneous tissue (m).

## 4. Discussion

Effects on ulcerative lesions were assessed by improvement according to the reduction in size of the lesions compared with untreated control mice. Cutaneous lesions in all infected female BALB/c mice started with redness and swelling at the site of inoculation post-infection. Swelling increased progressively onwards, with crust formation. Subsequently, cutaneous ulcers were developed and increased in size, then gangrene started to develop by the fourth weeks post-infection. The rate of spontaneous healing depends on several factors, including location of the lesion and the presence of secondary bacterial infections, which is in agreement with Ziaie and Sadeghian (2008) [33]. Infected, non-treated mice showed surface epithelial ulceration and localized dermal infiltrate composed of mixed acute and chronic non-specific inflammatory cellular infiltrates, which were composed of macrophages admixed with lymphocytes and neutrophils. Numerous *Leishmania amastigotes* were seen either inside or outside the macrophages causing congested blood vessels and leading to interference of the blood supply by the inflammatory cellular infiltration, and these results are in agreement with those of Maha et al., 2012 [34]. While sections taken from the skin of mice treated with allicin liquid and Cip showed an intact epidermis with remarkable reduction in inflammatory cells (Figure 5A,B).

The mechanism of the action of allicin effectiveness was attributed to the rapid reaction of thiol groups on a *L. major*. The most effective treatment included allicin and ciprofloxacin, which showed marked regression in the lesion size and improve lesion healing and parasite resolution in BALB/c mice co-infected with *L. major.*

Martins-Duarte et al. [35], mentioned that the in vivo studies showed that Cipro derivative administration was well tolerated and did not result in serious toxicity to mice, and only mild toxicity was observed after treatment with high doses (200 mg/kg) of Ph-Cipro. While all mice treated with Cipro died by day 10 post-infection, some mice treated with Cipro derivatives remained alive for at least 60 days, suggesting that Cipro derivatives cured *T. gondii* infection in treated mice. Considering that we used an acute model of infection, the modest rate of mice survival (13–25%) after treatment with Cipro derivatives is not irrelevant.

Skin and soft tissue infections are second in prevalence only to gastrointestinal illnesses in these patients. Infections include ventilator-associated pneumonia, bacteremia, indwelling device-associated infection, peritonitis, biliary tract infection, ocular infections, pyonephritis, lumboperitoneal shunt infection, and surgical and burn wound infections, and infections have been associated with a high mortality rate [36,37,38,39,40,41,42]. Other putative virulence factors were identified without experimental evidence [43]. *Flavimonas oryzihabitans*, known previously as *Pseudomonas oryzihabitans*, and a member of the Centers for Disease Control (CDC) group Ve-2, is a Gram-negative organism that has rarely been implicated as a human pathogen [44].

*Flavimonas oryzihabitans* is a soil and saprophytic microorganism that can survive in moist environments and rice paddles [45]. Only seven reported cases of human infection have been caused by *Flavimonas oryzihabitans. Flavimonas oryzihabitans* and *Chryseomonas luteola* have been placed in CDC groups Ve2 and Ve1, respectively [44]. *Flavimonas oryzihabitans* was previously isolated from cases of bacteremia, central nervous system (CNS) infections, wound infections, peritonitis, sinusitis, catheter-associated infections in Acquired Immune Deficiency Syndrome (AIDS) patients, and pneumonia [44]. Thus, *Flavimonas oryzihabitans* can cause infections in individuals with immune-related diseases, such as leishmaniasis, leukemia, and AIDS.

*Aeromonas* species are opportunistic pathogens, entering through wounds or affecting only stressed or otherwise immunocompromised hosts [46]. *A. salmonicida* has been isolated from humans [47]. The genus *Aeromonas* belongs to the family Vibrionaceae. *Aeromonas hydrophila* is the most commonly isolated species associated with human disease. Soft tissue infection, gastrointestinal illness, meningitis, pneumonia, endocarditis, arthritis, and osteomyelitis/sepsis can also be caused by *Aeromonas* sp. [36].

Chryseobacteria have been described in infections of skin and soft tissue, wound infection, meningitis, pneumonia, endocarditis, bacteremia dialysis-associated peritonitis, abdominal abscesses, sepsis, ocular infections, bronchitis, sinusitis, epididymitis, and prosthesis-associated septic arthritis [48,49,50]. Newborns and immunocompromised hosts of all ages are infected primarily by *Chryseobacterium meningosepticum* [48]. Frequent isolates of *Stenotrophomonas maltophilia* from wounds and other skin lesions have also been reported [51,52,53,54,55]. Other manifestations of *Stenotrophomonas maltophilia* soft tissue infection include umbilical cellulitis [56], prepatellar bursitis [57], cat scratches, and human bite wounds [55]. *Stenotrophomonas maltophilia* has been reported in the United States to cause health care-associated infections and ranks second among Gram-negative pathogens in the National Nosocomial Infection Surveillance System [58].

*Pseudomonas aeruginosa* contributes substantially to wound-related morbidity and mortality worldwide. The organism enters the blood, causing sepsis that can spread to the skin and lead to ecthyma gangrenosum, a black necrotic lesion [16]. It then produces several substances that are thought to enhance its colonization and infection of host tissue [59].

In humans, *Lactococcus lactis* subsp. *lactis* has been associated with endocarditis [60] and has also been isolated from clinical samples of blood, skin lesions, and urine [61]. The most frequent non-neoforman cryptococcal species reported to cause human disease is *Cryptococcus albidus*, a saprophytic fungus frequently found in the air or on the skin of healthy patients [62].

Allicin, one of the active components of freshly crushed garlic homogenates, has a variety of antimicrobial activities. The pure form of allicin exhibits antibacterial activity against a wide range of Gram-negative and Gram-positive bacteria, including the multidrug-resistant genera *Escherichia*, *Salmonella*, *Staphylococcus*, *Streptococcus*, *Klebsiella*, *Proteus*, *Bacillus*, and *Clostridium*, which are all affected by various garlic preparations. In addition, acid-fast bacteria, such as *Mycobacterium tuberculosis*, are sensitive to garlic [63]. The resistance mechanism of some strains to garlic is not clear, but some authors assume that the mucoid layers or hydrophilic capsule could prevent allicin from penetrating into the bacteria; however, this requires additional in-depth investigation [23].

From the above results, Cip 5 appeared to exhibit the strongest effect on the bacterial isolates tested, whereas the least effective antibiotic was LZD 30. The bacterial strain that exhibited the highest resistance to antibiotics was *Stenotrophomonas maltophilia* with a rate of resistance of 73.33%. These results demonstrated that *Stenotrophomonas maltophilia* was resistant to multiple antibiotics. *Stenotrophomonas maltophilia* was resistant to different antibiotics and disinfectants [63,64], and high-level multidrug resistance was found in clinical isolates [64,65]. Multidrug-resistant strains are also readily selected from susceptible *Stenotrophomonas maltophilia* in the laboratory [65]. Recent evidence indicated that the intrinsic and acquired multidrug resistance of *Stenotrophomonas maltophilia* might include antibiotic efflux as a contributing factor [65].

*Burkholderia cepacia* and *Pseudomonas aeruginosa* followed *Stenotrophomonas maltophilia* in resistance to the tested antibiotics a resistance rate of 66.67% for the two bacteria strains. Major causes of infections in Western society result from *Pseudomonas aeruginosa* and could arise from high resistance to antibiotics. The high intrinsic resistance arises from a combination of unusually restricted outer-membrane permeability and secondary resistance mechanisms, such as energy-dependent multidrug efflux and chromosomally encoded periplasmic β-lactamase. Mutational resistance to most classes of antibiotics causes a high level of natural resistance to arise quickly [66].

Gram-negative bacteria exhibit on their outer membrane a lipopolysaccharide and phospholipid bilayer that contains proteins termed porins that form water-filled channels as the major conduit for the diffusion of hydrophilic molecules. The mechanism of porins in *Pseudomonas aeruginosa* is as follows: OprF comprises the major porin for larger compounds, such as tri- and tetra-saccharides and possibly antibiotics, and forms a majority of small channels and a minority of larger channels [67]. The small channels are most likely due to the N-terminal half forming an eight-stranded β-barrel with a small central water-filled channel that is too small to permit substrate uptake. *B. cepacia* complex bacteria are multidrug-resistant owing to innate and acquired mechanisms of resistance [68].

The antibiotic efflux mechanisms of *Pseudomonas aeruginosa* and *B. cepacia* are increasingly recognized major factors in the intrinsic and acquired resistance of a number of significant human pathogens, including bacteremia cases [67]. The same rate of antibiotic resistance (60%) was found between *Comamonas acidovorans*, *Erwinia* sp., and *Chryseomonas indologenes*. *Flavimonas oryzihabitans*, *Aeromonas hydrophila*, *Pantoea* spp., *Aeromonas salmonicida* subsp. *salmonicida*, and *Chryseomonas luteola* exhibited moderate antibiotic resistance 46.76%, 26.76%, 26.76%, 33.33%, 20%, and 20%, respectively. All *Aeromonas hydrophila* isolates exhibited multidrug resistance [69]. Several virulence factors are specific for *A. salmonicida*: the type three secretion system (T3SS) encoded on a large plasmid was described for the first time in the *Aeromonas* genus [70,71]; VapA, a protein of the surface layer [72]; a type I pilus [73]; three type IV pilus systems [74]; superoxide dismutases [73]; and some extracellular proteins, including serine protease (AspA) [75], glycerophospholipid:cholesterol acyltransferase (GCAT or SatA) [76], and several hemolysins (aerolysins) [77].

Several studies have focused on drugs interfering with sterol biosynthesis. One double-blind trial in Saudi Arabia investigated the clinical efficacy of topical 1% clotrimazole (Canesten, Bayer, Kiel, Germany) and 2% miconazole (Daktarin, McGregor Cory Ltd., Banbury, UK). Both treatment groups showed improvements, with clotrimazole being most effective [78]. Another study in mice examined the topical versus oral delivery of terbinafine and itraconazole in BALB/c mice infected with *L. major* [79].

## 5. Conclusions

Many pathogenic and nonpathogenic bacteria that accompany cutaneous leishmaniasis in mice were investigated for their possible contribution to skin deterioration. Control of pathogenic bacteria using the natural product allicin and some antibiotics, such as Cip 5, exhibited high response rates for halting the growth of and curing *L. major* and accompanying bacterial infections; these products might also exhibit an effect on the *L. major* parasite itself. Thus, allicin and ciprofloxacin could be promising for the treatment of human cutaneous leishmaniasis and the control of secondary bacterial infections.

## Figures and Tables

**Table 1:** Lesion size (mm) in mouse skin one and four weeks after infection and treatment.

Doses	First Week	Four Weeks	*p*-Value
	Mean ± SD	Mean ± SD	
Control (Infected, non-treated)	8.22 ± 0.88	10.8 ± 1.89	<0.0001
Liquid allicin (0.30 μM/mouse)	7.88 ± 1.67	5.65 ± 1.01	0.107
Ciprofloxacin (10 mg/mL)	7.43 ± 1.99	6.79 ± 1.16	0.775
Allicin (0.30 μM + Ciprofloxacin (10 mg/mL))	7.66 ± 1.97	3.17 ± 1.12	0.058 *

SD: Standard Deviation * Significant at *p* < 0.05.

**Table 2:** Percentages of Gram-positive and -negative microorganisms isolated from cutaneous leishmaniasis in skin ulcers of mice.

Microorganism	Gram Stain	Total Isolates (*n* = 48)	%
*Erwinia* sp.	−	2	4.16
*Pantoea* sp.	−	2	4.16
*Chryseomonas luteola*	−	4	8.33
*Stenotrophomonas maltophilia*	−	2	4.16
*Pseudomonas aeruginosa*	−	2	4.16
*Burkholderia cepacia*	−	4	8.33
*Aeromonas salmonicida* subsp. *Salmonicida*	−	4	8.33
*Aeromonas hydrophila*	−	2	4.16
*Flavobacterium indologenes*	−	2	4.16
*Chryseobacterium meningosepticum*	−	4	8.33
*Bacillus* sp.	+	2	4.16
*Aerococcus viridans*	+	4	8.33
*Lactococcus lactis* subsp. *lactis*	+	2	4.16
*Chryseomonas indologenes*	−	2	4.16
*Flavimonas oryzihabitans*	−	6	12.5
*Comamonas acidovorans*	−	2	4.16
*Cryptococcus albidus*	+	2	4.16
Gram-positive	+	10	20.84
Gram-negative	−	38	79.16

**Table 3:** The effect of liquid allicin at 50 µL (disc diffusion) on bacterial isolates as a zone of inhibition (mm).

Bacterial Isolates	Allicin Zone of Inhibition (mm)
*Aeromonas salmonicida* subsp. *salmonicida*	15
*Pantoea* sp.	15
*Flavimonas oryzihabitans*	−
*Erwinia* sp.	−
*Chryseomonas indologenes*	10
*Chryseobacterium meningosepticum*	12
*Stenotrophomonas maltophilia*	−
*Burkholderia cepacia*	10
*Pseudomonas aeruginosa*	10
*Burkholderia cepacia*	10
*Bacillus* sp.	15
*Chryseomonas luteola*	10
*Cryptococcus albidus*	15
*Comamonas acidovorans*	−
Efficiency rate (%)	71.43

**Table 4:** Antimicrobial resistance of 24 h cultured bacterial isolates based on the development of inhibitory zone diameters after application of discs containing specific antimicrobial agents.

%	E	CFR	TE	C	K	CIP	CT	SXT	LZD	F	VA	TIC	N	AMP	AML		Antibiotic
Resistance	15	30	30	30	30	5	25	25	30	300	30	75	30	25	25		
	≤13	ND	≤11	≤12	≤13	≤15	ND	≤10	≤20	≤14	≤13	≤11	≤13	≤13	≤13	R	Microorganism
	14–22		12–14	13–17	14–17	16–20		11–15	21–22	15–16	14–18	12–14	14–15	14–16	14–17	I	
	≥23		≥15	≥18	≥18	≥21		≥16	≥23	≥17	≥19	≥15	≥16	≥17	≥18	S	
60	R	R	15	15	15	30	15	25	R	R	R	R	R	R	R		*Comamonas acidovorans*
0	17	30	15	15	30	30	16	30	30	24	30	30	15	30	30		*Chryseobacterium meningosepticum*
0	25	30	20	18	30	30	10	25	24	20	20	30	20	30	20		*Bacillus*
60	R	R	13	R	20	30	13	R	R	R	R	12	15	R	R		*Erwinia* sp.
26.67	16	ND	25	15	25	25	10	R	R	R	R	30	20	20	20		*Pantoea* sp.
26.67	15	20	24	18	20	30	20	30	R	26	R	12	16	R	R		*Aeromonas salmonicida* subsp. *salmonicida*
20	14	R	20	R	R	20	10	30	30	18	20	25	16	26	25		*Chryseomonas luteola*
60	16	R	14	30	R	30	R	30	R	R	15	R	R	R	R		*Chryseomonas indologenes*
46.67	14	R	20	20	R	R	R	15	R	R	14	12	R	14	18		*Flavimonas oryzihabitans*
66.67	R	R	R	15	20	30	12	20	R	R	R	R	R	R	R		*Burkholderia cepacia*
66.67	R	R	12	14	R	30	15	25	R	R	R	R	R	R	R		*Pseudomonas aeruginosa*
33.33	R	12	12	15	20	20	14	20	R	15	R	13	20	R	R		*Aeromonas hydrophila*
73.33	R	R	R	20	R	25	14	R	R	R	R	R	R	R	R		*Stenotrophomonas maltophilia*
	6	7	2	2	5	1	2	3	10	8	8	5	6	8	8	Resistance number for each antibiotic	
	46.15	53.8	15.39	15.39	38.46	7.70	15.38	23.08	76.92	61.54	61.54	38.46	46.15	61.54	61.5	Resistance rate (%)	
	83.85	30.7	84.61	84.61	61.54	92.30	84.61	76.92	23.08	38.46	38.46	61.54	83.85	38.46	38.4	Sensitivity rate (%)	

Mean zones of inhibition for common antibiotics tested: ≥18 mm (S = sensitive), 13–17 mm (I = intermediate), <13 mm (R = resistant), except noted above and ND = not detected: treated as a common antibiotics inhibition zone. AMP 25 = ampicillin (25 μg), AML 25 = amoxicillin (25 μg), N 30 = neomycin (30 μg), TIC 75 = ticarcillin (75 μg), VA 30 = vancomycin (30 μg), F 300 = nitrofurantoin (300 μg), LZD 30 = linezolid (30 μg), SXT 25 = sulfamethoxazole trimethoprim (25 μg), CT 25 = colistin sulfate (25 μg), Cip 5 = ciprofloxacin (5 μg), K 30 = kanamycin (30 μg), C 30 = chloramphenicol (30 μg), TE 30 = tetracycline (30 μg), CFR 30 = cefadroxil (30 μg), and E 15 = erythromycin (15 μg).
